# Safety of brigatinib following alectinib-induced-pneumonitis: Case report

**DOI:** 10.1016/j.rmcr.2024.102160

**Published:** 2024-12-27

**Authors:** Blerina Resuli, Heidi Galarza, Laura Elsner, Diego Kauffmann-Guerrero, Jürgen Behr, Amanda Tufman

**Affiliations:** a Department of Medicine V, LMU University Hospital, LMU Munich, Germany; b Comprehensive Pneumology Center Munich (CPC-M), German Center for Lung Research (DZL), Germany

**Keywords:** Alectinib, Brigatinib, NSCLC, Pneumonitis, Case report

## Abstract

Anaplastic lymphoma kinase tyrosine kinase inhibitors (ALK TKIs) show robust efficacy and has revolutionized the treatment of NSCLC patients harboring an ALK-rearrangement. Side effects, sometimes even serious such as pneumonitis, can occur with ALK TKIs.

We report a case of a patient with ALK positive advanced NSCLC who developed pneumonitis during treatment with first-line alectinib. With no alternative etiology of pneumonitis identified, the patient was treated with corticosteroids and discontinuation of alectinib. Following rapid clinical recovery and radiographic resolution of the opacities, the patient was started with brigatinib, with no recurrence of the clinical symptoms or radiographic findings of pneumonitis. While further descriptions are needed, our experience suggests that switching to a second ALK-TKI may be a safe therapeutic option in some patients who develop drug-induced pneumonitis on ALK TKIs.

## 1 Introduction

Anaplastic lymphoma kinase (ALK) inhibitors for oncogenic ALK gene rearranged non-small cell lung cancer (NSCLC), noted in 3.6–4.4 % of patients with NSCLC [1]. ALK TKIs have changed the treatment paradigm and improved the prognosis of oncogenic addicted NSCLC patients [2].

However adverse events (AEs) associated with ALK TKIs inevitably occur and cannot be ignored. The incidence of serious adverse events (SAEs) ranges from 27.1 to 45.7 % in patients receiving treatment with ALK TKIs [3]. Among these SAEs, pneumonitis is a rare but hazard AE that can be life threating and its incidence has gradually increased in recent years. The incidence of pneumonitis in current data varies from 0.4 % to 0.9 % among different studies [4]. Timely diagnosis of ALK-TKIs is key to improving the prognosis of these patients. However, no reports were available regarding the utility of switching to brigatinib after alectinib-induced pneumonitis. Here we report a case of a patient with NSCLC EML4-ALK translocation with safely tolerated brigatinib following alectinib-induced pneumonitis.

## 2 Case presentation

A 65-year-old Caucasian, never smoker women with a history of bipolar disorder in medical treatment with lithium was diagnosed in December 2022 with a mass in the left superior lung measuring >4 cm with invasion of mediastinum and satellite nodules in the same lobe. Positron Emission Tomography/Computed Tomography (PET/CT) scan showed additional hypermetabolic activity of the lung lesion as well as in the contralateral lung and right hilar and mediastinal lymph nodes. Magnetic resonance image (MRI) of the brain revealed a frontal lesion measuring 4,1× 4,9 cm concerning for metastasis. Biopsy of the lung mass was consistent with a TTF1 positive, lung adenocarcinoma with a tumor proportion score of programmed death-ligand 1 (PD-L1) of 3 %. Next-generation sequencing (NGS) revealed an echinoderm microtubule-associated protein-like 4-anaplastic lymphoma kinase (EML4-ALK) fusion.

**Fig. 1:**
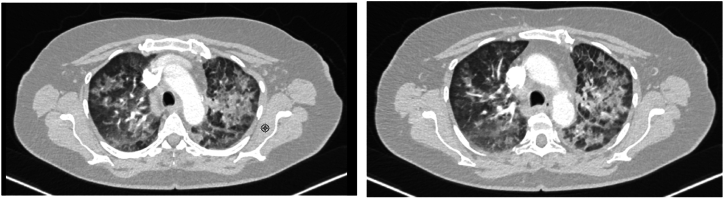
CT images of the chest: diffuse ground-glass opacities involving the entirety of both lung fields.

**Fig. 2:**
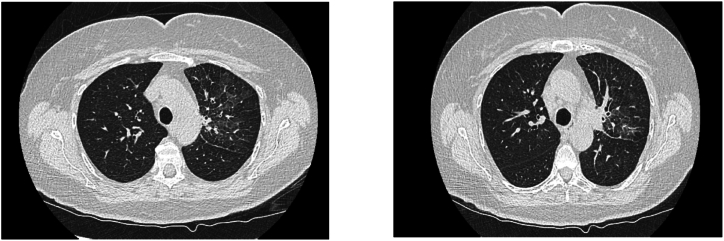
CT images of the chest after 3 weeks while the patient remained on corticosteroids and off alectinib, the previously ground-glass opacities were resolved.

In January 2023 following the diagnosis of stage IV (cT4cN3cM1c) ALK-rearranged NSCLC the patient was started first-line alectinib 600 mg twice daily. After 2 months of alectinib treatment the patient reported dyspnea and chest pain and diffuse ground-glass opacities were found in the CT scan of the chest (Fig. 1). Sputum was negative for bacterial infection. Alectinib was discontinued at this time and the patient initiated Prednisolone 1 mg/kg/daily for grade 2 pneumonitis. Within 48 hours, the patient ‘s clinical condition rapidly improved. Three weeks after initiating steroids a CT scan of the chest showed that the ground-glass opacities were almost completely resolved without evidence of short-term tumor progression (Fig. 2). MRI of the brain revealed a partial response.

Our multidisciplinary tumor board decided after recovery of pneumonitis, not to start alectinib at a low dose but to start brigatinib 90 mg daily for the first 7 days (beginning April 2023) and then increased to 180 mg once daily. Brigatinib was well tolerated, and the patient developed a partial response at the CT scan of the chest and abdomen after 6 weeks with a duration of response of 9 months without clinical or radiographic evidence of pneumonitis.

In January 2024 a CT scan of the chest and abdomen as well as the MRI of the brain revealed progression in the lung and lymph nodes and new lesions in the brain. In February 2024 following a careful discussion of the potential risks and alternative, the started lorlatinib 100 mg daily. She developed asymptomatic hyperlipidemia managed with statins without lorlatinib dose reduction. Under lorlatinib the patient did not develop any central nervous system (CNS) effects, despite the previous diagnosis of bipolar disorder. Lithium treatment and psychiatric monitoring were continued. The first on treatment imaging at 6-weeks revealed a partial response and the patient is still continuing lorlatinib 100 mg daily.

## 3 Discussion

To our knowledge, the safety of brigatinib in patients with a history of pneumonitis on other TKISTiasnot been previously reported. Although the incidence of pneumonitis is low, it can be life threatening and is incidence has gradually increased in recent years. Currently available data regarding the incidence of pneumonitis do not seem conclusive. The incidence of pneumonitis varies from 0,4 % to 0,9 % among different studies [4,5]. Once pneumonitis occurs, dose reduction, treatment suspension or medication discontinuation is required, which cab adversely affect OS and curative intention. Timely diagnosis and early treatment of pneumonitis caused by ALK-TIs are key to improving the prognosis of these patients. Alternative ALK TKIs are commonly used in clinical practice as different ALK TKIs may have different mechanisms for the development of pneumonitis [8].

Because of the mortality associated with drug-induced pneumonitis the question to whether or not to restart ALK targeted therapy in patients who have a history of pneumonitis requires careful consideration. A Limited number of previous case reports have described the successful of crizotinib, ceritinib, alectinib or brigatinib in patients with ALK-rearranged NSCLC who have recovered from pneumonitis secondary to ALK-TKIs [6,7,9,10]. In some of these cases patients resumed ALK-TKI after having mild pneumonitis. In comparison our patient, developed acute pneumonitis under alectinib and the subsequent tolerance on brigatinib was notable. Lorlatinib would have been an alternative choice for the treatment after alectinib, however; in light of the patient's psychiatric history and possible psychiatric side effects of lorlatinib, we chose brigatinib. It is reassuring that the subsequent treatment with lorlatinib did not cause psychiatric deterioration.

## 4 Conclusion

We describe a case of well tolerated brigatinib after alectinib-induced pneumonitis. Further research is needed to determine whether re-challenging with ALK TKIs carries an increased risk of recurrent pneumonitis. However, caution and close monitoring must be employed when considering this approach in clinical practice.

## CRediT authorship contribution statement

**Blerina Resuli:** Writing – review & editing, Writing – original draft, Validation, Resources, Project administration, Methodology, Investigation, Formal analysis, Data curation, Conceptualization. **Heidi Galarza:** Visualization, Validation. **Laura Elsner:** Visualization, Validation. **Diego Kauffmann-Guerrero:** Writing – review & editing, Visualization, Validation. **Jürgen Behr:** Visualization, Validation. **Amanda Tufman:** Writing – review & editing, Visualization, Validation, Supervision, Conceptualization.

## Data availability

The authors confirm that the data supporting the findings of this study are available within the article. Raw data that support the findings of this study are available from the corresponding author, upon reasonable request.

## Ethics statement

The study was conducted in accordance with the guideline form the ethic committee of the LMU University Hospital in Munich and it was conducted in accordance with the Declaration of Helsinki.

## Declaration of competing interest

The authors declare that they have no known competing financial interests or personal relationships that could have appeared to influence the work reported in this paper. No competing interests or relationships were declared by the authors.
